# The effectiveness of catgut implantation at acupoints for allergic rhinitis: A protocol for a systematic review and meta-analysis

**DOI:** 10.1097/MD.0000000000018554

**Published:** 2019-12-27

**Authors:** Juan Zhong, Yifeng Shen, Shuqin Liu, Menglin Dai, Yepeng Yang, Dazheng Zhang, Min Liu, Lijuan Zhang, Qinxiu Zhang

**Affiliations:** a Hospital of Chengdu University of Traditional Chinese Medicine, Otolaryngology Department/Radiology Department/MRI room; b School of Medical and Life Sciences/Reproductive & Women-Children Hospital, Chengdu University of Traditional Chinese Medicine, Chief medical officer's office, Chengdu; c Chengdu University of Traditional Chinese Medicine, School of Optometry; d Dujiangyan Medical Centre, Otolaryngology Department, Dujiangyan; e China Qingcheng Medical Research Laboratory of Traditional Chinese Medicine.

**Keywords:** catgut implantation at acupoints, allergic rhinitis, nondrug therapy, clinical trial, systematic review, protocol

## Abstract

**Background:**

Catgut implantation at acupoint (CIAA) is increasing used in allergic rhinitis therapy, and many studies have published that it is effective in the treatment of allergic rhinitis. However, it is controversy if CIAA can provide an evidence-based clinical benefit in the allergic rhinitis population.

**Methods:**

We will go through 8 databases, and conduct a systematic review of CIAA and health-related outcomes in allergic rhinitis patients according to the Preferred Reporting Items for Systematic Reviews. The primary objective is to assess the impact of Preferred Reporting Items for Systematic Reviews and Meta-Analyses (PRISMA) guidelines on clinical outcomes relevant to allergic rhinitis patients, such as effective rate, life-quality evaluation, and adverse events. Cochrane Risk Assessment Tool will be used to assess the quality of eligible studies according to the Cochrane handbook.

**Results:**

The results of this systematic review will provide a synthesis of current evidence of CIAA and we have a specific opportunity to determine the efficacy and safety of it.

**Conclusion:**

This study will explore whether or not CIAA can be used as one of the nondrug therapies to prevent or treat allergic rhinitis.

**PROSPERO registration number:** CRD42018095074 (https://www.crd.york.ac.uk/PROSPERO/#recordDetails)

## Abstract 2

Supplemental Digital Content is available in the text

## 1 Introduction

Allergic rhinitis (AR) is a disease induced by immunoglobulin E-(IgE)-mediated sensitization to environmental allergens. It poses a global health problem. Over 25% of Swedish citizens had been affected today,^[1]^ with evidence suggesting that the prevalence of the disorder is increasing.^[2]^ A study on epidemiologic analysis of allergic diseases in primary and middle school students in Foshan city of China showed that the prevalence of AR is 13.97%.^[3]^ Because of considerable symptomatic (nasal discharge, sneezing, nasal itching, and congestion) burdens, AR claims a high toll on patients’ lives, and negatively impacts patients’ quality of life (QoL),[^4^,^5^,^6^,^7^,^8^,^9^] this may include performance at work and school,[^10^,^11^] and can be associated with poor sleep quality,^[12]^ mood impairment,^[13]^ and even the ability to drive.^[14]^

Immunotherapy (subcutaneous injection, sublingual, or oral) are conventional treatment modalities for those boring symptoms, but the effectiveness of the therapies is in doubt and remains to be demonstrated conclusively.^[15]^ Hence, alternative nontherapy had been chosen for many.

Catgut implantation at acupoints (CIAA) is a special type of acupuncture that inserts medical threads (e.g., catgut or polyurethane PDO-RRB) into subcutaneous tissue or muscles at specific points (e.g., traditional acupuncture points or tender points).^[16]^ It may produce a strong and long-lasting therapeutic effect compared with acupuncture. This may because of the persistent stimulus to acupoints. CIAA has been particularly effective in treating chronic diseases and used for centuries to treat AR in China.^[17]^

To investigate the immunomodulatory effects of CIAA, Yang et al^[18]^ catgut for AR-related specific acupoints in rats, a shift from Th2 to Th1 occurs after CIAA treatment, especially around 2 weeks. And those suggest that CIAA can effectively reduce allergy symptoms and inflammatory parameters in the rat model of AR.

Even though 1 previous meta-analysis has been published in 2014,^[19]^ the efficacy and safety of CIAA on AR is still controversial. What is more, several RCTs[^20^,^21^,^22^,^23^] have been performed to evaluate the clinical benefits of CIAA in the treatment of AR recently. Therefore, to declare this issue, include newly published RCTs and update evidence-based result is extremely urgent.

## 2 Methods

This study had been registered at PROSPERO. The registration number is CRD42018095074. This meta-analysis will be based on the Preferred Reporting Items for the Systematic review and Meta-analysis of the (PRISMA) project.^[24]^

### 2.1 Inclusion criteria for study selection

#### 2.1.1 Type of studies

All the RCTs to explore the specific efficacy and safety of CIAA in the treatment of AR will be included. Cross-trials, quasi-RCT, case reports, observation study, animal study, repeatedly published studies, and studies did not have access to complete data will be excluded. If we are unable to find at least 5 eligible RCTs for the systematic review, we will broaden our inclusion criteria to include semi-randomized control studies, nonrandomized studies of CIAA in patients with AR using the Cochrane Effective Practice and Organization of Care (EPOC) approach to categorize the types of studies.^[25]^

#### 2.1.2 Types of participants

Participants who meet the diagnostic criteria of AR, either presenting with seasonal AR or perennial AR were all included. However, AR merged with allergic asthma or allergic conjunctivitis and other allergic diseases were excluded. This was done because targeted drug combination methods in these studies could not be used to compare the effects. All included participants in this review regardless of their age, race, and gender.

#### 2.1.3 Types of interventions and controls

We will only include studies which interventions involved CIAA with conventional medicine or placebo regimens. However, studies that compare the efficacy of different forms of CIAA will be excluded. Interventions considered for experimental groups vs control groups were as follows:

1. CIAA vs conventional medicine
2. CIAA combined with conventional medicine vs conventional medicine
3. CIAA combined with other complementary therapies vs other complementary therapies
4. CIAA vs placebo or no therapy
5. CIAA vs pseudo-catgut implantation therapy or no therapy

We excluded studies or trials with CIAA performed as a part of complex interventions versus other types of regimens, for example, CIAA plus another herbal medicine formula vs acupuncture therapies.

#### 2.1.4 Types of outcome measures

The primary outcome is the clinical effective rate. The secondary outcomes mainly including outcomes such as visual analog score (VAS), QoL inventory for nasal conjunctivitis, other symptoms scoring system results as recommended by guidelines,^[26]^ and adverse events.

### 2.2 Search methods for identification of studies

#### 2.2.1 Data sources

PubMed, Embase (Excerpta Medical Database), The Cochrane Library, the Chinese Cochrane Centre's Controlled Trials Register platform, the Wanfang Chinese Digital Periodical and Conference Database, the China National Knowledge Infrastructure (CNKI) database, and the VIP Chinese Science and Technique Journals Database will be researched by our author for relevant literature.

#### 2.2.2 Searching other resources

Chinese Clinical Trial Registry Center will also be screened for ongoing trials. We will also review the references of included manuscripts to identify any information about missed trials. We will contact the author if we cannot clearly identify information from the data.

#### 2.2.3 Search strategy

We will employ a broad electronic search strategy in Supplemental Digital Content (Appendix A).

### 2.3 Data extraction, quality, and validation

#### 2.3.1 Study selection and inclusion

Researchers will import the literature retrieved to the Endnote X7 and eliminate the duplicate data. All titles and abstracts returned using the search strategy above will be screened by 2 independent investigators (MLD, YPY) in line with our advanced inclusion criteria. And then, the full text of the entire study will be reviewed by 3 authors for analysis. Any differences will be resolved by consensus. Finally, another study member will resolve the inconsistencies and check the final literature that will be included.

#### 2.3.2 Data extraction and management

**Figure 1:**
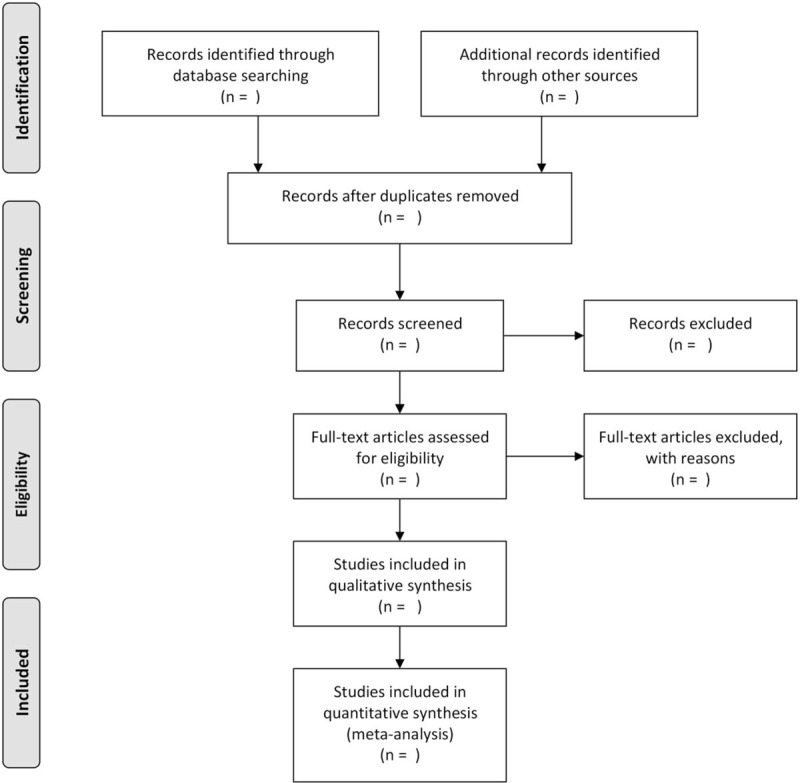
Flow diagram of study selection process.

The raw data from the papers will be extracted separately by 3 authors and will include: author details, publication information, sample size, and original study design information, such as intervention and comparison (dose, route, and time), outcome measures, and follow-up information. Catgut brand information will be also extracted from us if possible. All extracted data will be verified by a second investigator to ensure accuracy and completeness. All outcome variables will be collected, regardless of the number of studies that the outcome assessed. If conflict, arbitration will be conducted through discussion or through the 3rd reviewer (DZZ, ML, and LJZ). Preferred Reporting Items for Systematic Reviews and Meta-Analyses (PRISMA) diagram (Fig. 1) based on the search strategy and eligibility assessment to show the flow of included and excluded studies will be developed by us (DZZ, ML).

#### 2.3.3 Assessment of risk of bias

The methodologic quality of the included RCTs will be assessed based on the instrument developed in the Cochrane Handbook for Systematic of Interventions by three investigators. The tool evaluates studies based on 7 criteria: randomization generation, allocation concealment, blinding of outcome assessors, blinding patients/study personnel, incomplete outcome data (i.e., lost to follow-up), selective outcome reporting, and other risks of bias. We will define other bias as trials which may be sponsored by CIAA manufacturers, and in which baseline characteristics are not similar between the different intervention groups. We will also assess publication bias by examining funnel plots if there are 10 or more trials reporting the primary outcomes.

### 2.4 Quantitative data and statistical methods

#### 2.4.1 Quantitative data synthesis

Review Manager (RevMan) software version 5.3 will be applied to pool our data to perform the meta-analysis. Measurements of dichotomous data will to be expressed as relative risks along with 95% confidence intervals (CIs); for continuous data, mean difference, 95% CIs will be adopted, and *P* < .05 will be defined as statistically significant.

#### 2.4.2 Assessment of heterogeneity

In our review, *I* ^2^ values will be used to assess interstudy heterogeneity. When *I* ^2^ > 75%, considerable heterogeneity will be conformed, whereupon a random effects model will be applied. We will pool trials when the intervention form of those studies is adequately similar. Specific subgroups will be analyzed according to similar intervention forms or similar design (Fig. 1 PRISMA diagram).

#### 2.4.3 Assessment of reporting bias

If a sufficient number of studies are available (at least 10 studies), we will attempt to assess publication bias using a funnel plot.

#### 2.4.4 Subgroup analysis and investigation of heterogeneity

If there is a significant heterogeneity in the included trials, we will conduct subgroup analysis based on the type of disease, differences in treatment frequencies and follow-up duration will also be included.

#### 2.4.5 Sensitivity analysis

If the test for heterogeneity *P*-value is <.1 after performing the subgroup analysis, the sensitivity analysis will be conducted to evaluate the robustness of our results. The meta-analysis will be repeated after omitting the low-quality studies. Moreover, we will also assess whether the statistics model (random-effects model and fixed-effects model) will affect the current results.

#### 2.4.6 Grading the quality of evidence

We will apply the Grading of Recommendation Assessment, Development and Evaluation (GRADE) method to evaluate the level of confidence in regards to outcomes. Two independent reviewers will conduct the assessment. In most cases, disagreements were resolved by discussion. If disagreement remained after discussion, a 3rd reviewer will be consulted before taking the final decision on the disagreement.

## 3 Discussion

Result of the previous meta-analysis showed that catgut implantation was proved with only limited evidence for the treatment of AR. Robust RCTs with high quality and larger sample size in this field are hoped to be carried out in the future. Several RCTs[^20^,^21^,^22^,^23^] have been performed to evaluate the clinical benefits of CIAA in the treatment of AR recently. Therefore, promoting CIAA in the clinical treatment of AR with its acceptability is badly needed.

Moreover, we foresee several potential limitations with this systematic review: heterogeneity of clinical outcomes, substandard quality of existing studies, which are the focus of our project. Therefore, we will present our findings using descriptive methods, if necessary. This study protocol has been designed according to conventional acupuncture therapy for treatment of AR based on the study data or outcomes from existing published (and nonpublished) literature. Our hope is that the dissemination of this protocol will allow us to obtain feedback and constructive criticism of the methods before our study is conducted.

In conclusion, the proposed systematic review will provide insight into the clinical impact of CIAA in treatment of AR patients. The results have the potential to inform national and international guidelines on the care and management of CIAA in the AR population. The review will also help to highlight areas requiring further rigorously designed research on this topic.

## Author contributions

**Visualization and software:** Juan Zhong, Yifeng Shen.

**Data curation:** Shuqin Liu.

**Formal analysis:** Menglin Dai, Yepeng Yang.

**Methodology:** Dazheng Zhang.

**Project administration:** Min Liu.

**Software:** Juan Zhong, Qinxiu Zhang.

**Supervision:** Lijuan Zhang.

**Validation:** Menglin Dai.

**Visualization:** Shuqin Liu.

**Writing – original draft:** Juan Zhong.

**Writing – review & editing:** Yifeng Shen.

Juan Zhong orcid: 0000-0002-7805-0185.

## Supplementary Material

Supplemental Digital Content
