# Supplementary material for: The effectiveness of catgut implantation at acupoints for allergic rhinitis: A protocol for a systematic review and meta-analysis
Source: Medicine (Baltimore). 2019 Dec 27;98(52):e18554. doi: 10.1097/MD.0000000000018554 (PMC6946449; doi:10.1097/MD.0000000000018554)
Supplement: Supplemental Digital Content [file medi-98-e18554-s001.doc]

The search strategy will be:

#1. ("catgut"[MeSH Terms] OR "catgut"[All Fields]) AND ("embryo implantation"[MeSH Terms] OR ("embryo"[All Fields] AND "implantation"[All Fields]) OR "embryo implantation"[All Fields] OR "implantation"[All Fields]) AND ("acupuncture points"[MeSH Terms] OR ("acupuncture"[All Fields] AND "points"[All Fields]) OR "acupuncture points"[All Fields] OR "acupoints"[All Fields])

#2. ("acupuncture points"[MeSH Terms] OR ("acupuncture"[All Fields] AND "points"[All Fields]) OR "acupuncture points"[All Fields] OR "acupoint"[All Fields]) AND ("catgut"[MeSH Terms] OR "catgut"[All Fields]) AND embedding[All Fields]

#3. Thread[All Fields] AND embedding[All Fields] AND ("acupuncture"[MeSH Terms] OR "acupuncture"[All Fields] OR "acupuncture therapy"[MeSH Terms] OR ("acupuncture"[All Fields] AND "therapy"[All Fields]) OR "acupuncture therapy"[All Fields])

#4. #1~#3/OR

#5. "rhinitis, allergic"[MeSH Terms] OR ("rhinitis"[All Fields] AND "allergic"[All Fields]) OR "allergic rhinitis"[All Fields] OR ("allergic"[All Fields] AND "rhinitis"[All Fields])

#6. Rhinallergosis[All Fields]

#7.AR

#8. #5~#7/OR

#9. "Randomized controlled trial"[Publication Type] OR "randomized controlled trials as the topic"[MeSH Terms] OR "randomized controlled trial"[All Fields] OR "randomised controlled trial"[All Fields]

#10.#4 AND #8 AND #9
